# A novel insight on SARS-CoV-2 S-derived fragments in the control of the host immunity

**DOI:** 10.1038/s41598-023-29588-8

**Published:** 2023-05-17

**Authors:** Thais Sibioni Berti Bastos, André Guilherme Portela de Paula, Rebeca Bosso dos Santos Luz, Anali M. B. Garnique, Marco A. A. Belo, Silas Fernandes Eto, Dayanne Carla Fernandes, Fausto Klabund Ferraris, Leticia Gomes de Pontes, Tábata Takahashi França, Leonardo José Gil Barcellos, Flavio P. Veras, Pamela Bermejo, Giovanna Guidelli, Carla Maneira, Fellipe da Silveira Bezerra de Mello, Gleidson Teixeira, Gonçalo Amarante Guimarães Pereira, Bianca H. Ventura Fernandes, Paulo R. S. Sanches, Helyson Lucas Bezerra Braz, Roberta Jeane Bezerra Jorge, Guilherme Malafaia, Eduardo M. Cilli, Danilo da Silva Olivier, Marcos Serrou do Amaral, Renata J. Medeiros, Antonio Condino-Neto, Luciani R. Carvalho, Glaucia M. Machado-Santelli, Ives Charlie-Silva, Jorge Galindo-Villegas, Tárcio Teodoro Braga

**Affiliations:** 1 Department of Pathology, Federal University of Parana, Curitiba, Brazil; 2 Graduate Program in Biosciences and Biotechnology, Instituto Carlos Chagas, Fiocruz-Parana, Brazil; 3 Department of Cell Biology, Institute of Biomedical Sciences, University of São Paulo, São Paulo, Brazil; 4 Brasil University, Descalvado, São Paulo, Brazil; 5 Center of Excellence in New Target Discovery (CENTD) Special Laboratory, Butantan Institute, São Paulo, Brazil; 6 Center of Innovation and Development, Laboratory of Development and Innovation, Butantan Institute, São Paulo, Brazil; 7 Veterinarian, São Paulo, Brazil; 8 Department of Pharmacology and Toxicology, Oswaldo Cruz Foundation, FIOCRUZ, Rio de Janeiro, Brazil; 9 Laboratory of Human Immunology, Department Immunology, Institute Biomedical Sciences, University São Paulo, São Paulo, Brazil; 10 Laboratory of Fish Physiology, Graduate Program of Bioexperimentation, University of Passo Fundo, Santa Maria, Brazil; 11 Graduate Program of Pharmacology, Federal University of Santa Maria, Santa Maria, Brazil; 12 Center of Research in Inflammatory Diseases, Ribeirão Preto Medical School, University of Sao Paulo, Ribeirão Preto, São Paulo, Brazil; 13 Department of Pharmacology, Ribeirao Preto Medical School, University of São Paulo, Ribeirao Preto, São Paulo, Brazil; 14 Laboratório de Genômica e bioEnergia (LGE), Institute of Biology - Unicamp, Campinas, Brazil; 15 Department of Genomics, Faculty of Biosciences and Aquaculture, Nord University, Bodø, Norway; 16 Laboratório de Controle Genético e Sanitário, Diretoria Técnica de Apoio ao Ensino e Pesquisa, Faculdade de Medicina da Universidade de São Paulo, São Paulo, Brazil; 17 Instituto de Química, Universidade Estadual Paulista, Araraquara, SP Brazil; 18 Department of Physiology and Pharmacology, School of Medicine, Federal University of Ceará, Fortaleza, CE Brazil; 19 Biological Research Laboratory, Goiano Federal Institute, Urutai Campus, Urutaí, GO Brazil; 20 Integrated Sciences Center, Federal University of Tocantins, Araguaína, TO Brazil; 21 Institute of Physics, Federal University of Mato Grosso do Sul, Campo Grande, MS 79070‐900 Brazil; 22 Laboratory of Physiology, INCQS/Fiocruz Zebrafish Facility, Department of Pharmacology and Toxicology, National Institute for Quality Control in Health, Rio de Janeiro, Brazil; 23 Laboratory of Cellular and Molecular Biology, Department of Cell and Developmental Biology, Institute of Biomedical Science, University of Sao Paulo, University of São Paulo, São Paulo, Brazil; 24 Department of Pharmacology, University of São Paulo-ICB/USP, São Paulo, Brazil

**Keywords:** Biological techniques, Immunology

## Abstract

Despite all efforts to combat the pandemic of COVID-19, we are still living with high numbers of infected persons, an overburdened health care system, and the lack of an effective and definitive treatment. Understanding the pathophysiology of the disease is crucial for the development of new technologies and therapies for the best clinical management of patients. Since the manipulation of the whole virus requires a structure with an adequate level of biosafety, the development of alternative technologies, such as the synthesis of peptides from viral proteins, is a possible solution to circumvent this problem. In addition, the use and validation of animal models is of extreme importance to screen new drugs and to compress the organism's response to the disease. Peptides derived from recombinant S protein from SARS-CoV-2 were synthesized and validated by in silico, in vitro and in vivo methodologies. Macrophages and neutrophils were challenged with the peptides and the production of inflammatory mediators and activation profile were evaluated. These peptides were also inoculated into the swim bladder of transgenic zebrafish larvae at 6 days post fertilization (dpf) to mimic the inflammatory process triggered by the virus, which was evaluated by confocal microscopy. In addition, toxicity and oxidative stress assays were also developed. In silico and molecular dynamics assays revealed that the peptides bind to the ACE2 receptor stably and interact with receptors and adhesion molecules, such as MHC and TCR, from humans and zebrafish. Macrophages stimulated with one of the peptides showed increased production of NO, TNF-α and CXCL2. Inoculation of the peptides in zebrafish larvae triggered an inflammatory process marked by macrophage recruitment and increased mortality, as well as histopathological changes, similarly to what is observed in individuals with COVID-19. The use of peptides is a valuable alternative for the study of host immune response in the context of COVID-19. The use of zebrafish as an animal model also proved to be appropriate and effective in evaluating the inflammatory process, comparable to humans.

## Introduction

Severe acute respiratory syndrome coronavirus 2 (SARS-CoV-2) was first reported in China in December 2019 and identified as the causative virus of Coronavirus 2019 (COVID-19), a disease that would later become a pandemic^1^. This virus infects the target cells by binding its spike (S) glycoprotein, present on the viral surface, to the angiotensin-converting enzyme 2 (ACE2) receptor^2^. This interaction occurs more specifically through the receptor-binding domain (RBD), present in the S1 subunit of the S protein, to the ACE2 receptor, with consequent proteolytic cleavage of the S2 subunit, which bring together the viral and cellular membranes^3,4^.

Vascular endothelial cells and alveolar macrophages express ACE2 and are among the first target cells of viral entry^5^. In response to the invading agent, the immune system develops an inflammatory state to try to stop the replication and spread of the virus^6^. In the early stages of a viral infection, there is production of type I interferon (IFN-α/β), which has direct antiviral activity, and activation of NF-κB, which induces the transcription of pro-inflammatory cytokines, which attract other cells to mount the adaptive immune response^7,8^. Infection results in the accumulation of immune cells, such as macrophages and monocytes, which release the pro-inflammatory cytokines interleukin-1 β (ilib), tumor necrosis factor α (tnfa), interleukin-6 (il6), and interferon- γ (ifng1) as a response against the virus^9^. A dysregulated inflammatory response of the immune system triggers an excessive release of these mediators, triggering cytokines storm and generating severe local and systemic pathologies in the individual^10^.

To better understand the pathophysiology of diseases and develop new treatments, in vitro approaches, organoids and animal models are widely used tools, mainly due to the advances in genetic engineering techniques and molecular biology^11,12^. An ideal animal model for the study of COVID-19 should possess the ACE2 receptor in adequate levels and organ-specific expression of it, should be susceptible to infection, support viral replication, and exhibit the classic symptomatology characterized by metabolic and histological changes upon infection^13,14^, in addition to presenting other signs/symptoms and behavioral changes analogous to those observed in humans. However, no animal model that have been used, such as mice^15^, syrian hamsters^16^, ferret^17^, mink^18^, and non-human primates^19^, present all these characteristics.

In addition to traditional host models, zebrafish (*Danio rerio*), an extremely versatile and attractive organism, have gained prominence in research 10.3389/fphar.2020.00680. Some studies have already been using zebrafish as a model to study COVID-19 once the animal has the main components used for viral entry into the cell, such as the receptor Ace2, TMPRSS2, the proteases cathepsin L, trypsin and furin^20–24^. Zebrafish larvae virus-infected from inoculation into the swim bladder showed modest viral replication^25^. Alternatively, when the virus was added to the water of larvae, the animals showed reduced expression of Ace2, increased expression of ilib and tnfa, and reduced il4/il13b production, characterizing the induction of a significant antiviral and pro-inflammatory immune response^26^. Recent study evaluated the induced hyperinflammation in zebrafish larvae via the Tlr2/Myd88 signaling pathway induced by the spike protein in zebrafish^27^.

In the present research, we evaluated the effects of S-derived peptides due to their potential use as immunizant agents. We observed that the SARS-CoV-2 S protein fragments were able to interact with immune system receptors and ACE2 in a stable manner, triggering an increase in mortality, in addition to the development an inflammatory process with macrophage recruitment and changes in the polarization dynamics of these cells. Changes in biomarkers of cellular stress in these larvae were also evaluated.

## Results

### Zebrafish can be used to studies of SARS-CoV-2

**Figure 1:**
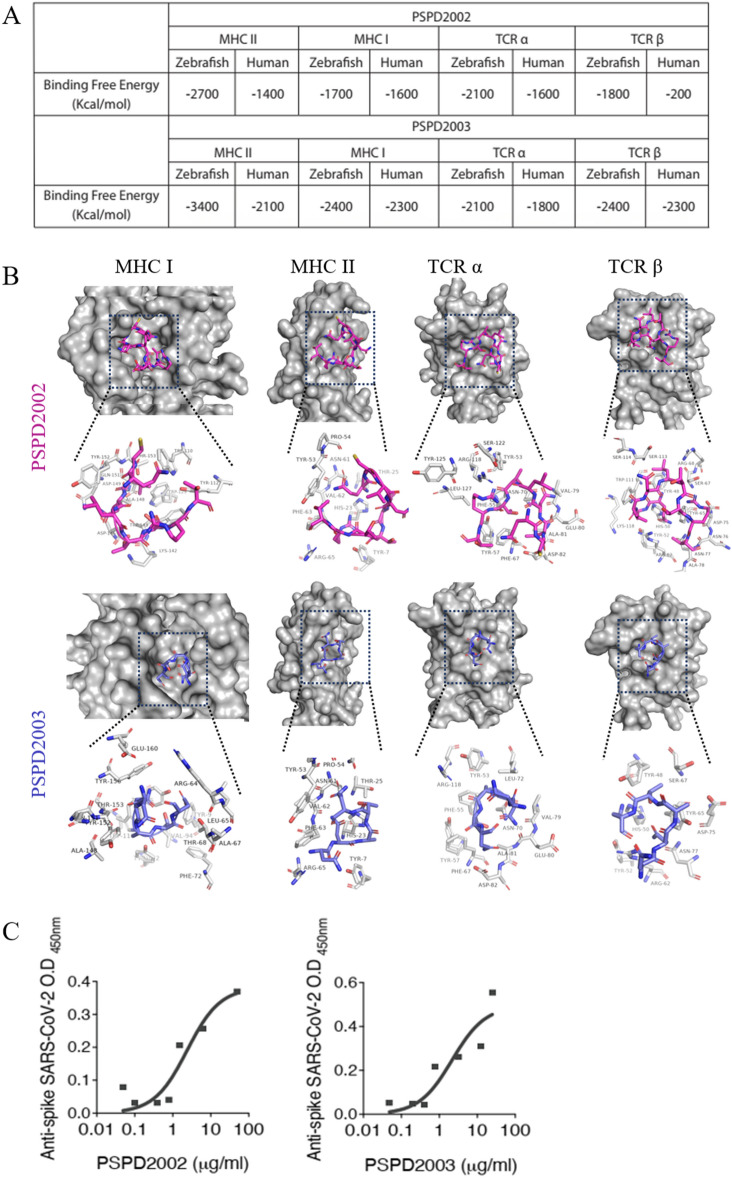
Peptides PSPD2002 and PSPD2003 potentially interact with receptors of the human and zebrafish immune systems. (**A**) Calculated interaction free energy (Kcal/mol) for PSPD2002 and PSPD2003 interactions for MHC II, MHC class I, TCRα and TCRβ in *Homo sapiens* and *Danio rerio* homologous receptors. (**B**) Docking analysis highlighting the interaction sites between PSPD2002 (purple) and PSPD2003 (blue), and the MHC II, MHC I, TCRα and TCRβ ligands of *Danio rerio*. Serial dilutions of the peptides being recognized by anti-Spike SARS-CoV-2 antibody for evaluation of the specificity of PSPD2002, in (**C**), and PSPD2003, in (**D**).

Initially, we performed a computational analysis of the protein–protein interaction prediction of SARS-CoV-2 Spike-derived peptides in humans and zebrafish. For this, we used a sequence of protein S (residues 16 to 165) and generated two peptides using the virtual proteolytic cleavage as a pattern memorizing phagolysosomal proteolysis. Two peptides named PSPD2002 (residues 16 to 22) and PSPD2003 (residues 121 to 125) have been chosen due to their promising antigenic potential. Both PSPD2002 and PSPD2003 present the binding free energy in protein:ligand interactions for class I MHC, class II MHC, TCRα and TCRβ similar in both *Homo sapiens* and *D. rerio* homologous receptors (Fig. 1A). Docking analysis supports the similarity of the PSPD2002:ligand and PSPD2003:ligand interaction at the receptor-binding site for class I MHC, class II MHC, TCRα and TCRβ in humans and fish (Fig. 1B).

In order to validate both PSPD2002 and PSPD2003 as SARS-CoV-2 specifically derived peptides, we quantified them, at several dilutions, using anti-spike SARS-CoV-2 IgG. We observed that PSPD2002 and PSPD2003 are virus-derived and quantifiable in vitro and are properly recognized by the anti-spike SARS-CoV-2 specific antibody (Fig. 1C,D respectively). Altogether, we observed that the peptides PSPD2002 and PSPD2003 potentially interact with receptors of the human and zebrafish immune systems and are therefore a usable alternative for vaccine or target druggable strategies due to their promising antigenic potential.

### Peptide of SARS-CoV-2 Spike stably bind to the ACE2 in zebrafish

**Figure 2:**
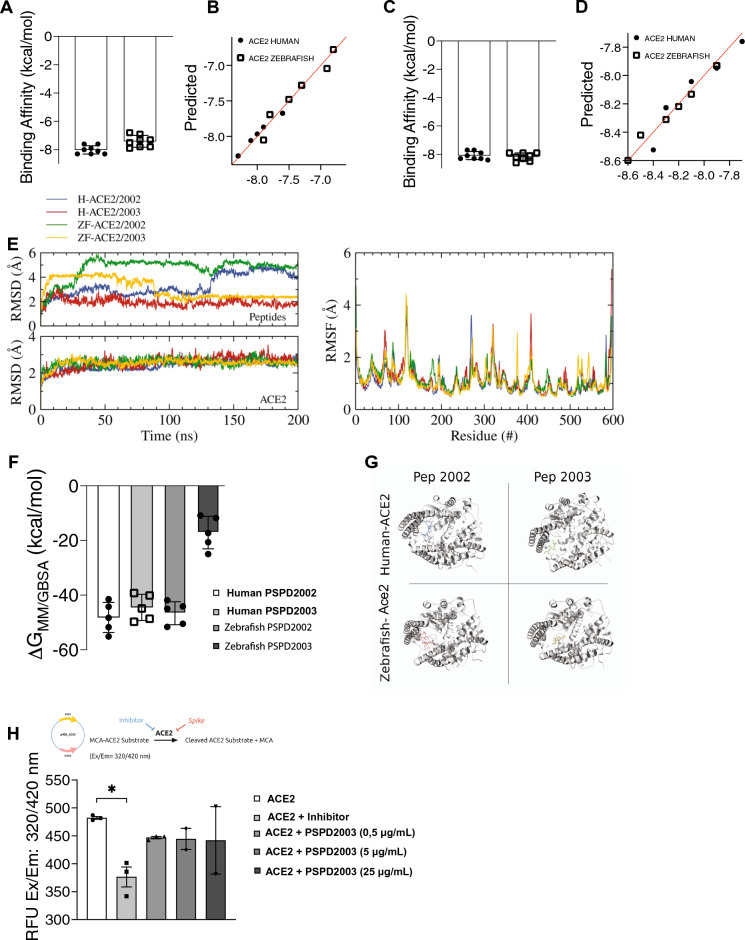
Affinity and binding stability between S-derived peptides and the ACE2 receptor. (**A**) and (**C**) Molecular dynamics analysis of interaction strength between PSPD2002 (**A**) or PSPD2003 (**C**) and ACE2 receptor of human and zebrafish, and predictive values for PSPD2002 (**B**) or PSPD2003 (**D**). (**E**) Analysis of the convergence and stability of the interaction of proteases over 200 ns for peptides (top) and ACE2 (bottom), from RMSD (root-mean-square deviation) analysis (left). Fluctuation analysis of the different residues of the proteases using RMSF (root-mean-square fluctuation) docking (right). (**F**) Analysis of the binding free energy during 100 ns of stimulation, between both peptides (PSPD2002 and PSPD2003) and the human and zebrafish Ace2 receptors. (**G**) Graphical representation showing the interaction regions between the PSPD2002 and PSPD2003 fragments with the proteases of the quaternary structure of the human and zebrafish Ace2 receptors. (**H**) Expression of ACE2 in *Saccharomyces cerevisiae* strain. The analysis of the catalytic activity of the receptor against the synthetic inhibitor (control) and different concentrations of the peptides was quantified from the Relative Fluorescence Unit (RFU) read in excitation range of 320 nm and emission range of 420 nm (Ex/Em: 320/420). Å: angstrom, a unit of length equivalent to 0.1 nm. MM/GBSA: Molecular mechanics generalized Born surface area. *p < 0.05.

To confirm the stability of the S-derived fragments binding to ACE2 we performed a molecular dynamics analysis of both PSPD2002 and PSPD2003 with ACE2. They presented acceptable affinity data, both above the mean of strong interactions (− 6.0 kcal/mol). The affinity values for PSPD2002 and human ACE2 was − 8.01 ± 0.29 kcal/mol and zebrafish Ace2 was − 7.41 ± 0.41 kcal/mol (Fig. 2A,B). The affinity values for PSPD2003 and human ACE2 was − 8.08 ± 0.28 kcal/mol and zebrafish Ace2 was − 8.17 ± 0.27 kcal/mol (Fig. 2C,D). To evaluate the stability over time of the PSPD2002 and PSPD2003 peptides with ACE2, we performed stability analysis of the simulated systems. To access the convergence and stability of the RMSD and RMSF simulations, plots were done for all the systems (Fig. 2E). The RMSD plots for the peptides indicate a fluctuation over time depending on simulated system. PSPD2002 peptide is bigger that PSPD2003 and presented more fluctuations over time when compared to the shorter peptide, PSPD2003. The same plot for the ACE2 of human and zebrafish presented the convergence of the proteases, indicating high stability over time. The RMSF plot shows the displacement of the residues of ACE2 proteases. Results for all the systems overlaps with minor intensity differences among them. The low values indicate the residues are stable and did not significantly change during the MD simulations, while the peaks indicate regions with poor secondary structure stability, like loops and turns. The radius of gyration (RoG) and surface area completes the stability analysis showing that the proteases were stable over time. RoG shows if the proteases increased or decreased its size over time. For all the simulations the RoG were around 25.3 Å, indicating stability (Fig. S1—left) (Fig S1—left). The surface area of the proteases were also stable over time, with minor fluctuations for the zebrafish/2003 complex (Fig. S1—left) (Fig S1—right).

MM/GBSA energy calculation were done over the last 100 ns of simulation. The average binding value were − 48.3 kcal/mol, − 44.9 kcal/mol and − 45.0 kcal/mol for the H-ACE2/PSPD2002, H-ACE/PSPD2003 and ZF-ACE2/PSPD2002 respectively (Fig. 2F). For the zebrafish Ace2 in the presence of the PSPD2003, the energy value was around − 17.3 kcal/mol. The energy decomposition indicated that the peptides were in the same region for the systems H-PSPD2002, H-PSPD2003 and ZF-PSPD2002, while it was slightly different for the ZF-PSPD2003 (Fig. S2). The protease residues with negative values cooperates to bind the peptide, while the positive values indicate the protease residue does not cooperate to the binding. For the first three complexes the peptide bound in similar regions: between residues 60–100, 160–200 and 360–400. In these cases, the contribution of the protease residues changed in intensity and also the residues that were contributing to the binding. On the other hand, the ZF-PSPD2003 complex only shares the region of the residues between 160 and 200, with another contribution around the residues 480–500. This could be the reason for the large total binding difference between this system compared to the others. Representative structures for each simulated system show the peptides interacting with the human and zebrafish Ace2 protease (Fig. 2G).

Once we confirmed the stable form interaction of the peptides with the ACE2 receptor, we performed in vitro interaction between the peptides and human ACE2 using an ACE2-expressing *Saccharomyces cerevisiae* strain cell extract. For that, the coding human ACE2 sequence was cloned in vector p426 and further transformed in yeast BY4742. The recombinant *S. cerevisae* strain was used for whole-cell extract preparation and initially used to confirm the heterologous ACE2 catalytic activity (Fig. 2H). After the confirmation of such activity—the first available report on functional human ACE2 in yeast -, the same cell extract was used to confirm the binding effect of the peptides. The assay reveals that the fragment of Spike in the solution is capable of inhibiting ACE2 activity by − 1.1 fold (*p* value < 0.001). The same inhibitory effect is observed using a synthetic ACE2 inhibitor, used here as a control, confirming that the Spike protein developed for this study is effective (Fig. 2H). Together, these data show that the synthesized peptides are derived from regions of strong interaction of the SARS-CoV-2 S-protein with the ACE2 receptor.

### Peptide of SARS-CoV-2 Spike can be responsible for activating mammalian cells

**Figure 3:**
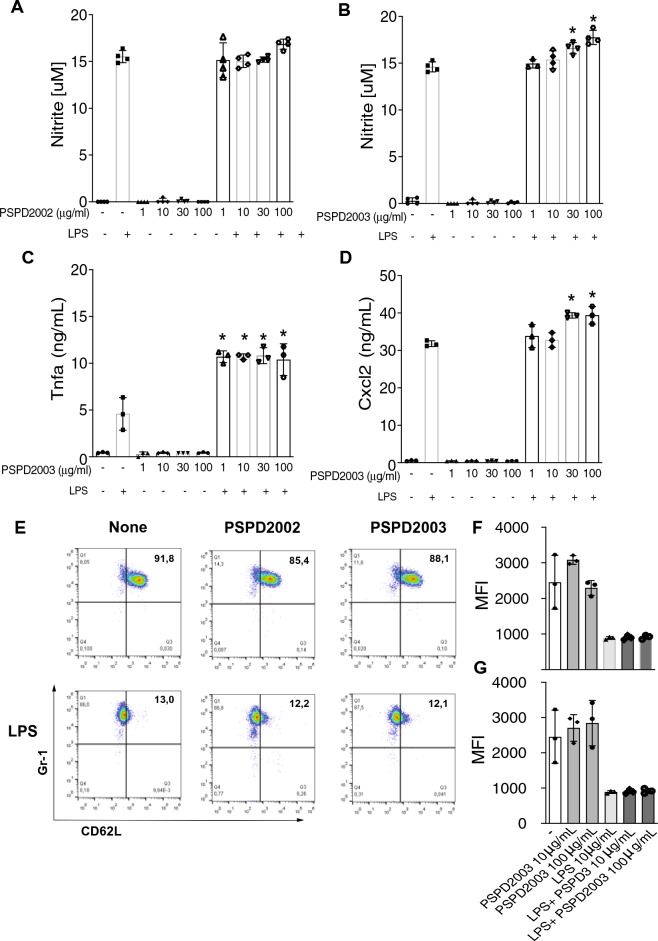
Effect of PSPD2002 and PSPD2003 peptides on mammalian cells. Quantification of nitric oxide (NO) production in AMJ2-C11 cells in the presence and absence of lipopolysaccharide (LPS), challenged with 1, 10, 30 and 100 ug/ml of PSPD2002 (**A**) and PSPD2003 (**B**). Quantification of Tnfa (**C**) and Cxcl2 (**D**), in AMJ2-C11 cells challenged with PSPD2003, in the presence and absence of LPS. **E** Neutrophils (GR1^+^ cells) activation status demonstrated by representative flow cytometry graphics of unsensitized (top row) or sensitized with LPS (bottom row) and challenged with PSPD2002 (middle column) or PSPD2003 (right column). The x-axis represents L-selectin (CD62L) marker. (**F**) and (**G**) Mean fluorescence intensity (MFI) quantification of cells unsensitized (**F**) or sensitized with LPS (**G**). All experiments were performed in triplicate in three independent days. All data are expressed as the mean ± standard deviation. *p* < 0.05 was considered to indicate a statistically significant difference.

Once the synthesis and interaction of the peptides were confirmed, we performed in vitro analyses by challenging mammalian immune cells with PSPD2002 or PSPD2003. We initially stimulated immortalized murine macrophage AMJ2-C11 with both peptides, in the presence or absence of lipopolysaccharide (LPS). We observed that despite no response upon PSPD2002 stimulus, PSPD2003 induced NO production in a dose response manner, as indicated by nitrite measurement (Fig. 3A,B). Moreover, PSPD2003 was able to induce Tnfa and Cxcl2 production (Fig. 3C,D). Human’s FACS-sorted blood circulating neutrophils were also stimulated with both PSPD2002 and PSPD2003 at 10 and 100 μg/ml in the presence or absence of LPS. However, regardless the presence of LPS, both peptides were unable to modulate CD62L on cell surface, an indicator of cellular activation profile^28,29^ (Fig. 3E–G). Together, these data reveal that the peptides were able to induce a pro-inflammatory response mediated mainly by macrophages, marked by increased NO, Tnfa and Cxcl2. Whereas neutrophils did not show an activation profile in response to the fragments.

### Peptide of SARS-CoV-2 Spike decreases survival rate and promotes histological changes in zebrafish larvae

**Figure 4:**
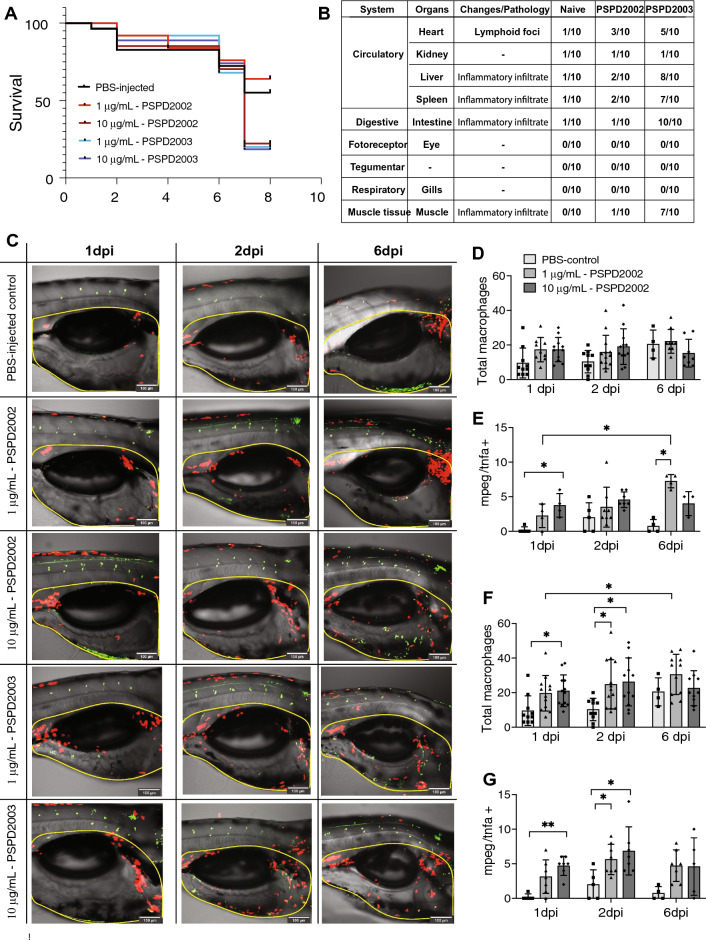
Effects of immunization of S-derived peptides on zebrafish larvae. (**A**) Kaplain-Meyer curve representing the survival rate (y-axis) over 10 days (x-axis) of larvae immunized with water, and with different concentration (1 or 10 µg/mL) of PSPD2002 and PSPD2003. (**B**) Table with the main histopathological changes observed in zebrafish larvae immunized with PSPD2002 and PSPD2003. Different organs from different systems were analyzed and classified according to the alterations/pathology in a score ranging from 0 to 10. (**C**) Representative images obtained in confocal microscope of transgenic Tg(*mpeg:mCherry/tnfa:eGFP*) larvae immunized by inoculation into the swim bladder with different concentrations of PSPD2002 and PSPD2003 at 1, 2 and 6 days post injection (dpi). Macrophages are marked by red fluorescence (mCherry) expression and tnfa by green fluorescent protein (GFP) expression. The region surrounded by the yellow dash represents the counting region. Lateral view in sagittal plane of the animal, with caudal-cranial orientation (left–right) and pelvic fin below, allowing visualization of the swim bladder (centered), intestinal tract (below), and notochord and muscle tissue above. Counting the total number of macrophages (mCherry+) (**D**) and double-labeled pro-inflammatory macrophages (mCherry + /tnfa +) (**E**) in larvae immunized with PSPD2002 at 1, 2 and 6dpi. Counting the total number of macrophages (mCherry +) (**F**) and dual labeled pro-inflammatory macrophages (mCherry + /tnfa +) (**G**) in larvae immunized with PSPD2003 at 1, 2 and 6dpi. Scale bar 100um in (**C**) From** C** to** G**, experiments were performed in three independent days and n vary from 4 to 12. All data are expressed as the mean ± standard deviation. *p < 0.05; **p < 0.01.

To use zebrafish as a model to understand COVID-19 pathophysiology, we analyzed the effects of PSPD2002 and PSPD2003 at 1 and 10 μg/ml after their injection in the larvae swim bladder at 6 days post fertilization (dpf). Animals immunized with 10 μg/ml of PSPD2002 and with 1 μg/ml or 10 μg/ml of PSPD2003 had a lower survival rate (*p* < 0.05) than the water-injected control group. When comparing mortality between the different peptides at 1 μg/ml concentration, animals exposed to PSPD2003 died more than the group exposed to PSPD2002 (*p* < 0.05). The mortality rate did not differ in the comparison between the water-injected control group and the group injected with 1 μg/ml PSPD2002 (Fig. 4A). The histopathological changes in several organs were also analyzed and it was observed an intense inflammatory infiltration upon immunization mainly with PSPD2003 peptide in many tissues. Markedly, liver, spleen, intestine, and muscle obtained from PSPD2003-injected fishes showed an intense inflammatory infiltrate (Fig. 4B). In this regard, we observed that the fragments were able to trigger death in zebrafish larvae, as well as histopathological changes, similarly to what is observed in individuals with COVID-19.

### A role of macrophage recruitment and activation upon S-derived peptides injection

We then hypothesized that macrophage recruitment and activation could be controlled in the context of S-derived peptides injection. To address this hypothesis, we immunized larvae swim bladder at 6 dpf from transgenic animals Tg(Mpeg1:mCherry/TNFα:GFP), in which pro-inflammatory macrophages are represented by double positive cells, while non-inflammatory ones are mCherry^+^GFP^−^. After inoculation of PSPD2002 and PSPD2003, animals were evaluated under a confocal microscope on days 1, 2 and 6 following the procedure (Fig. 4C–G). Animals immunized with PSPD2002, did not show an increase in the total number of macrophages on the three different days (Fig. 4D). However, when analyzing the number of inflammatory macrophages in these animals, an increase was observed in the group exposed to 10 μg/ml as early as 1-dpi, when compared to the water-injected control group, while the group exposed to 1 μg/ml only demonstrated such increase at 6-dpi (Fig. 4E). Animals immunized with 1 μg/ml of PSPD2002 also showed a progressive increase in inflammatory cells, with more cells at 6-dpi than 1-dpi (Fig. 4E). Like PSPD2002, PSPD2003 triggered changes in the profile of these cells, being the group exposed to 10 μg/ml demonstrating increased numbers of double positive cells at 1- and 2-dpi (Fig. 4G). Injection of PSPD2003 at 1 μg/ml also led to increased inflammatory cells at 2-dpi (Fig. 4G). Different from PSPD2002 inoculation, PSPD2003 led to elevated total number of macrophages. When compared to the control group, animals injected with 10 μg/ml PSPD2003 showed a higher total number of these cells on day 1 and 2-dpi (*p* < 0.05) (Fig. 4F). Animals exposed to 1 μg/ml also showed an overall increase in these cells when compared to the control group, but only on the second day (*p* < 0.05). In this group a time-dependent response occurred, with more cells at 6-dpi than 1-dpi. Collectively, these data suggest that higher concentrations of the peptides can induce a faster inflammatory response with a peak up to 2 days after immunization and consequently with faster resolution as well. Additionally, it was seen that PSPD2003 induces a more pronounced inflammatory response in comparison with the PSPD2002 peptide.

### Oxidative stress biomarkers in larvae zebrafish

**Figure 5:**
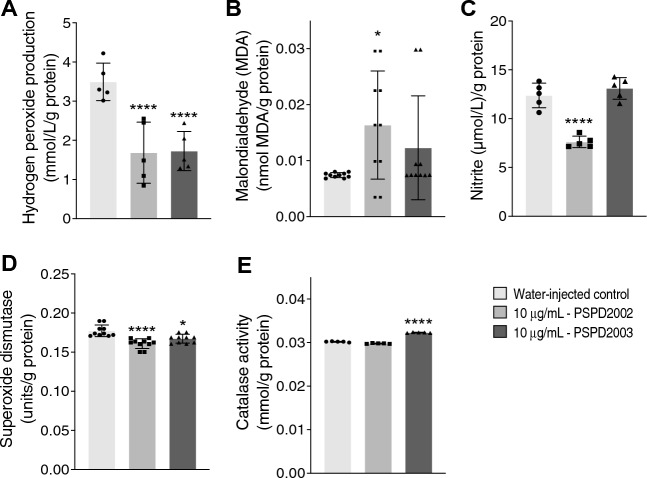
Changes in biomarkers of oxidative stress in zebrafish larvae immunized with 10 ug/ml of PSPD2002 or PSPD2003. The production levels of the oxidant compounds: hydrogen peroxide (**A**), malondialdehyde (**B**), nitrite (**C**), and of the antioxidants: superoxide dismutase (**D**) and catalase (**E**) were quantified. Experiments were performed in three independent days and n vary from 5 to 10. All data are expressed as the mean ± standard deviation. *p < 0.05; ****p < 0.0001 in the comparison with water-injected control.

Once demonstrating the macrophage dynamic throughout the time, the oxidant compounds hydrogen peroxide, malondialdehyde, nitrite, and the activities of antioxidant enzymes superoxide dismutase (SOD) and catalase (CAT) were also evaluated upon 10 μg/ml of peptides injection at 6- dpi. Despite the diminished production of hydrogen peroxide upon PSPD2002 and PSPD2003 injection (Fig. 5A), both peptides led to increased malondialdehyde production (Fig. 5B). The nitrite production, in turn, was reduced after PSPD2002 and unchanged after PSPD2003 injection (Fig. 5C). Changes were also observed in the antioxidant responses, i.e. the levels of SOD were reduced after PSPD2002 and PSPD2003 injection (Fig. 5D). CAT levels, on the other hand, was increased upon PSPD2003 injection, remaining unchanged after the PSPD2002 injection (Fig. 5E). Altogether, these data point to changes in the oxidative and antioxidative profile upon S-derived peptides injection. Moreover, there is a correlation between the degree of innate immune cells infiltration and the redox profile of the larvae during the SARS-CoV-2-derived peptides. Docking analysis revealed that all interactions of PSPD2002 and PSPD2003 with antioxidant enzymes showed acceptable affinity data, negatively exceeding the allowable docking score limitation (− 6.0 kcal/mol) (BRAZ et al. 2020). The affinity values for PSPD2002 were SOD (− 7.2 kcal/mol) and CAT (− 6.7 kcal/mol). For PSPD2003, affinity values were SOD (− 6.6 kcal/mol) and CAT (− 7.2 kcal/mol). All these data together suggest that fragments synthesized from the SARS-CoV-2 S protein are capable of immunizing animal model in vivo and they represent therefore a new safer alternative to trigger the inflammatory clinical signs characteristic of COVID-19.

## Discussion

Here, we demonstrate a novel role for macrophage-activation signaling in orchestrating the recruitment process in response to S protein. Although much effort has been made to understand the basis of molecular activation of macrophages, the mechanisms of SARS-CoV-2 S protein fragments by which macrophages mediate signaling are poorly documented in COVID-19. We identified peptides of SARS-CoV-2 Spike, with special role of PSPD2003, as key components to control the host immunity by macrophage recruitment and activation and inflammatory modulation in vitro and in vivo. Within the decrease in inflammatory macrophages, at later in the inflammatory process, we observed less Mpeg1^+^/TNFα^+^ cells, decreased SOD activity and consequently less hydrogen peroxide produced. Such data is further supported by an increase in catalase activity after PSPD2003 injection. Several studies report that ROS modulation and SOD and CAT substrates, superoxide ion and hydrogen peroxide, respectively, participate in cellular signaling in favor of proper inflammatory resolution^30–32^.

Among the imminent challenges in combating the pandemic is the intense search to develop adequate and reliable animal models that can reproduce the pathophysiology of the disease. The ideal model should be permissive to infection, allowing the study of the virus-host interaction and mimicking the clinical pathology of the disease^33^. Our study brought a new alternative to study the inflammatory process triggered in a patient with COVID-19 by using synthetic peptides in a zebrafish model. The peptides PSPD2002 and PSPD2003, synthesized from the S protein of SARS-CoV-2 were also validated by in silico. They showed binding stability over time and interaction with both human and zebrafish immune system receptors and ACE2. This interaction is extremely important, because unlike the application of peptides based on their antiviral activity aimed at developing new drugs^34^, our proposal is to use these particles to challenge and mimic the organism's response to the virus. But the restricted handling of the SARS-CoV-2 virus in a biosafety level 3 (BSL-3) laboratory has still been a major problem for its research. To circumvent this, at least some virus-like particles without virulence that can be handled in BSL-2 have been developed^35,36^ However, the use of peptides is even more advantageous for our purpose, since besides dispensing with the use of BSL-3, they present lower synthesis costs.

Besides the innovation in the use of peptides, we bring the use of an emerging animal model in zebrafish for the study of human diseases. This allows us to study the immune response to stimulation with viral particles, as well histopathological changes. Zebrafish have already been used to study infections with several viruses that trigger disease in humans such as dengue virus^37^, chikungunya virus^38,39^, zika^40^, herpes (HSV-1)^41,42^, cytomegalovirus^43^, influenza A^44,45^; hepatitis B^46^, and hepatitis C^47^. The hyperinflammation seen in patients with severe COVID-19 is markedly caused by an exacerbated response of the body to infection, which can lead to multiple organ failure and death. The leukocyte infiltrate in the lungs of these patients is mostly composed of macrophages^48^. In viral infection, the inflammatory cascade initiated by macrophages, such as the release of cytokines like IL-6 and IL-1β that act in the recruitment of neutrophils and cytotoxic T cells, contributes to both viral control and tissue damage^49^.

Understanding the role of the innate immune system during viral infections is particularly promising in the zebrafish model system, since the animal exclusively displays innate immunity in the first weeks of life^50^. Humanized adult zebrafish infected with SARS-CoV-2 protein S in the swim bladder showed accumulation of macrophages and granulocytes^9^. Animals infected with the recombinant spike protein also showed a toxic inflammatory response with macrophage infiltrate^51^. We found a similar response. The peptides were able to increase the recruitment of these cells to the inoculation site and modulate their phenotype. In animals immunized with PSPD2002, although there was no increase in the total number of these cells within days, a higher number of pro-inflammatory cells was observed. When administered the highest concentration 10 μg/ml of PSPD2002, this increase quickly occurred already at 1-dpi, while the peak of these cells in the group exposed to 1 μg/ml was later, being observed only at 6-dpi. Thus, the higher the peptide concentration, the faster the inflammatory process was established and resolved, while lower concentrations of the peptide triggered the slower response, progressively until day 6.

Animals immunized with PSPD2003 showed an increase in the total number of macrophages and a markedly pro-inflammatory phenotype. At 1 and 2-dpi, animals immunized with 10 μg/ml PSPD2003 showed a higher number of inflammatory macrophages when compared to the control group. While the 1 μg/ml administration only had its expressive increase in the total number of these cells at 6-dpi, but now with an antiinflammatory profile, characterizing the beginning of the resolution of the process. This time-response relationship and severity of the inflammatory process is observed in SARS-CoV-2 infected patients. Patients with higher viral titers had a shorter duration of illness, averaging 7 days, while patients with a lower viral load maintained the condition for 19 days^52^. In a study where SARS-CoV-2 spike protein was injected into the swim bladder of larvae, a high infiltration of inflammatory macrophages was observed at 4dpi, resulting in an inflammatory process. In 7dpi analysis no cells with an inflammatory profile were observed, but cellular debris was, which confirms the reduction of the inflammatory process^9^.

Studies indicates that pathogenicity of SARS-CoV-2 is associated with oxidative stress^53^. Oxidative stress is the result of an imbalance between oxidant production and antioxidant mechanisms and triggers the events that may contribute to the severity of COVID-19 in patients^54,55^. The complex responsible for the respiratory burst, important in the recruitment of additional phagocytes and ROS production, is conserved between humans and zebrafish^50^. In our study an increase in malondialdehyde (MDA) synthesis was observed by injection of both peptides. MDA is an indicative parameter of oxidative stress, which makes it possible to correlate this marker with viral infections of the respiratory tract, especially RNA viruses^56^. Nitrite synthesis was reduced after administration of the peptide PSPD2002. Plasma levels of nitrite are involved with regional endothelial NO synthase (eNOS) enzyme activity^57^. Viral infection triggers damage to the endothelium of the respiratory tract, leading to reduced eNOS and therefore resulting in reduced nitrite^58^. The respiratory burst performed by macrophages when exposed to virus infection leads to ROS production^59^. Patients infected with SARS-CoV-2, show high serum levels of antioxidant enzymes^60^. The enzymes SOD and CAT act in the neutralization of oxidant molecules, such as ROS^61^. It was observed that SOD levels decreased after administration of both peptides, and CAT levels increased after administration of the peptide PSPD2003. SARS-CoV-2 infection leads to excessive ROS production, which triggers a weakened antioxidant response^62^. This reduction in SOD activity has also been observed during infections with other viruses^63,64^.

We further evaluated the activation of neutrophils challenged with the peptides. In homeostasis, neutrophils show high expression of CD62L, but during inflammatory processes the expression of this selectin is reduced. Post-ICU patients on COVID-19 exhibited a pronounced increase in circulating CD62L^low^ neutrophils showing fragility and hypersecretion^65^. The elevated numbers of neutrophils in patients with severe COVID-19 accompanied by increased levels of neutrophil extracellular traps (NETs) were related to an increased risk of death and the development of lung injury and microthrombi^66^. In our study, the peptides were unable to activate neutrophils, marked by reduced expression of CD62L on their surface.

In conclusion, our study shows new alternatives for studying the characteristic inflammatory process observed in COVID-19 patients. Non-virulent peptides synthesized from SARS-CoV-2 protein S were able to immunize zebrafish larvae and trigger the inflammatory process, marked by macrophage infiltration and changes in oxidative stress biomarkers. The change in macrophage polarization in a dose-dependent manner triggered by the fragments can be extrapolated to the clinical context, where viral load is associated with patient clinical outcome. Our results are important in helping to elucidate the pathophysiology of the disease and characterize an animal model that allows broad drug screening, enabling the development of new therapies.

## Materials and methods

### Study strategy

All methods were performed in accordance with the relevant guidelines and regulations. Operationally we first performed in silico analyses for the synthesis of the SARS-CoV-2 S-protein-derived fragments. Once determined, these peptides were further evaluated by computational methodologies regarding their interaction with receptors of the immune system, both in humans and in zebrafish. Once confirmed these interactions and the high immunogenicity of the fragments, we performed in vitro experiments, using macrophages and neutrophils cell cultures to analyze inflammatory markers and activation of these cells. Then, finally, we injected these synthesized fragments into the swim bladder of zebrafish larvae to study survival, macrophage polarization and biomarkers of oxidative stress.

### Synthesis, purification, and characterization of peptides

The peptides PSPD2002 (Gln-Cys-Val-Asn-Leu-Thr-Thr-Arg-Thr-COOH; MW: 1035.18 g/mol) and PSPD2003 (Asn-Asn-Ala-Thr-Asn–COOH; MW: 532.51 g/mol) were obtained manually using the solid phase peptide synthesis method (SPFS) and purified by high-performance liquid chromatography (HPLC) with a reverse-phase column using different purification methods. The analysis of the synthesized peptides' identity was carried out in a mass spectrometer using the methodology described by Charlie-Silva et al. (2021)^67^.

### Expression of ACE2 in yeast

The physiological effect of the S-derived peptides on human ACE2 was assessed using an ACE2 activity assay kit (BioVision) and an ACE2-expressing yeast cell extract. A Saccharomyces cerevisiae BY4741 background was transformed with a plasmid containing the human ACE2 coding sequence (optimized for expression in yeast) cloned in the vector p426. Whole-cell extract was prepared according to Çağlayan and Wilson (2014)^68^ and the lysate was further used for the heterologous ACE2 functional analysis and the Spike effect on ACE2. For the last, 0.5 µg/ml of the S-derived peptides were used together with the cell extract, and a synthetic-specific ACE2 inhibitor as control. Procedures were performed as described by the kit manufacturer. Briefly, the protocol is based on the ability of an active ACE2 to cleave a synthetic MCA-based substrate to release a free fluorophore that is monitored by fluorescence at an excitation maximum of 320 nm and an emission maximum of 420 nm using a CLARIOstar microplate reader (BMG LABTECH). All assays were performed in triplicates.

### Molecular dynamics simulations setup

All simulations were carried out using AMBER18^69^. The atomic coordinates from the human and zebrafish with the PSPD2002 and PSPD2003 peptides were obtained from the molecular docking and prepared for Molecular Dynamics (MD) simulations as follows. The complexes were submitted to H++ web-server^70^ to set the amino acid protonation state at pH 7.4. The all-atom interaction was described using FF19SB^71^ force field. The complexes were embedded in octahedral boxes with edges, at least, 10 Å from the surface of the solute, and containing TIP3P water molecules and neutralized with Na + ions. To eliminate bad contacts from starting structures, each system was minimized in two steps. First, the energy minimization of the protein–ligand constrained complexes (force constant of 10.0 kcal/mol-Å2) were performed with 5000 steepest descent steps followed by 5000 conjugate gradient steps and by unconstrained energy minimization rounds (10.000 steps). After minimization, the complexes were gradually heated from 10 to 298 K or 310 K for, respectively, zebrafish or human complexes, during 500 ps under canonical (NVT) ensemble, while the protein was restrained with force constant of 10 kcal/mol-Å2. Subsequently, an equilibration step was performed using isothermal-isobaric (NPT) ensemble for 5 ns. Finally, the production runs 200 ns for each system, performed in NVT ensemble without any restraints. The temperature (298 K or 310 K) and pressure (1 atm) were controlled by Langevin coupling. The SHAKE constraints were applied to all bonds involving hydrogen atoms to allow a 2-fs dynamics time step. Long-range electrostatic interactions were calculated by the particle-mesh Ewald method (PME) using 8-Å cutoff^72^.

### Molecular dynamics analysis

The CPPTRAJ^73^ program of AmberTools19^74^ was used to analyze the MD simulations. Root mean square deviation (RMSD) and the radius of gyration (Rg) of Cα were calculated to determine the system quality and stability and to determine the equilibration and convergence of the systems. Protein flexibility was calculated by root mean square fluctuation (RMSF) for all Cα atoms, residue‐by‐residue over the equilibrated trajectories. In order to have a representative structure for the simulations, clustering analysis was performed with the k‐means method ranging from 2 to 6, and to access the quality of clustering, the Davies‐Bouldin index (DBI) values and silhouette analyses were used. The interaction energy between the ACE2 protein and the peptides were calculated using the generalized Born GB‐Neck2^75^ implicit solvent model. Molecular mechanics/generalized Born surface area (MM/GBSA) energy was computed between the protein and the peptides in a stable regime comprising the last 100 ns of the MD simulation, stripping all the solvents and ions.

### Peptide of SARS-CoV-2 Spike stably bind to the ACE2

The physiological effect of the Peptide of SARS-CoV-2 Spike on human ACE2 was performed using an ACE2 activity assay kit (BioVision) and ACE2-expressing yeast cell extract. In short, *Saccharomyces cerevisiae* strain BY4742 was transformed with a plasmid containing the human ACE2 coding sequence cloned in vector p426. Whole-cell extract was prepared using Çağlayan and Wilson (2014)^68^ protocol and the lysate was further used for the heterologous ACE2 functional analysis and Spike effect on ACE2 catalytic activity. For the last, 0.5 µg/ml of the peptide of SARS-CoV-2 were used together with the cell extract, and a synthetic-specific ACE2 inhibitor as control. Procedures were performed as described by the kit manufacturer. All assays were performed in biological triplicates.

### Zebrafish maintenance

Wild-type zebrafish from the AB line, and specific pathogen-free (SPF), were raised in Tecniplast Zebtec rack (Buguggiate, Italy). SPF were maintained in the zebrafish housing systems at Medicine Faculty of University of Sao Paulo, and Tg (Mpeg1:mCherry/TNFα:GFP) and AB zebrafish were maintained at Pathology Department of University of Parana facilities. Fish used for the experiments were obtained from natural crossings and raised according to standard methods. Zebrafish were kept in 3.5 L polycarbonate tanks and fed three times a day with Gemma micro by Skretting (Stavanger, Norway). The photoperiod was 14:10 h light–dark cycle and the water quality parameters were 28 °C ± 2 °C; pH = 7.3 ± 0.2; conductivity 500 to 800 µS/cm, referred to as system water. The procedures were approved by the Ethics Committee (CEUA) of the Medicine Faculty of University of Sao Paulo and registered under protocol number 1514/2020 and by the Ethics Committee (CEUA) of the University of Parana and registered under protocol number 1410. All procedures were performed following the Three R principle (Replacement, Reduction, and Refinement) and following the ARRIVE guidelines (Animal Research: Reporting of In Vivo Experiments).

### Macrophage’s function studies

The animals were selected at 5dpf according to the expression of the red fluorescent protein mCherry and the green fluorescent protein (GFP), confirming the lineage Tg (Mpeg1:mCherry/TNFα:GFP). Only doubly labeled animals were used in the experiment. The next day, at 6dpf, the peptides PSPD2002 and PSPD2003 were inoculated into the swim bladder. The peptides were synthesized and lyophilized, and then dissolved in water at 1 μg/mL and 10 μg/mL contractions. Two different groups, injected with water and unmanipulated animals, were kept as controls. Capillary tubes (Glass line, Precision, China) with 75 mm length, inner and outer diameter of 1 mm and 1.5 mm, respectively, and without heparin were used for microinjection. These capillaries were prepared in a puller following the established parameters respectively, of: heat 500, force of 70, and distance of 9.20. Microinjection was performed with a Pneumatic PicoPump PV830 microinjector (World Precision Instruments-WPI). After selection and microinjection, the embryos were placed in a 96-well microplate, with 1 embryo per well and incubated at 28 °C (Digital incubator, Sterilifer, Brazil). The solutions were renewed every 24 h. For selection, injection and microscopic analysis, the animals were previously anesthetized in tricaine methanesulfate (MS-222) solution. We performed 3 independent experimental exposures.

### Confocal microscopy imaging

After microinjection of peptides into the swim bladder, tracking of the leukocyte was performed on a Nikon A1R MP + Confocal microscope. The fluorescent protein mCherry was excited using 543 nm and detected in the typical red detection channel at 550–650 nm. GFP was excited using 488 nm and detected a typical green detection channel at 505–550 nm. The intensity used for both lasers was 5.00. Such images were generated in high resolution, in a configuration of 1 frame every 4 s and 1024 resolution and acquired at 5 μm intervals between the different planes. After obtaining the images, they were evaluated in ImageJ Fiji software (NIH), where the number of macrophages labeled with red fluorescence only (mCherry), or double labeled with both red and green fluorescence (GFP) were manually counted.

### Histology from multiple organs

Fixation and decalcification of the adult zebrafish for histology and immunofluorescence was performed according to Moore et al. 2002^76^ For histopathological analysis, 5-μm-thickness sections were mounted on slides and dewaxed in an oven at 60 °C and hydrated in decreasing solutions of xylol three times, and once in xylol + alcohol, for 10 min each, followed by a 100, 90, 80, and 70% alcohol battery and washed with distilled water for five minutes. They were then stained with hematoxylin and eosin for observation of the general cellular structures.

### Oxidative stress biomarkers in zebrafish larvae

The effects peptide of SARS-CoV-2 Spike on oxidative stress reactions were evaluated based on indirect nitric oxide (NO) determination based on REDOX regulated processes via nitrite measurement^77^, on thiobarbituric acid reactive species (TBARS) predictive of lipid peroxidation^78^, production of reactive oxygen species (ROS) and on hydrogen peroxide (H_2_O_2_), which plays an essential role in responses to oxidative stress in different cell types^79^. The Griess colorimetric reaction^80^ was used to measure NO. This reaction consisted in detecting nitrite resulting from NO oxidation. TBARS levels were determined based on procedures described by Ohkawa et al.^81^ with adaptations for conduction in microtubes and ELISA microplate reading. The reagent of 1,1,3,3-tetraethoxypropane was used as a standard solution in the reaction with thiobarbituric acid (TBA) reactive substance. In brief, the principle of this method depends on the determination of the pink color which is produced by the interaction of TBA with (malondialdehyde) MDA. Hydrogen peroxide and ROS production, on the other hand, were evaluated using the methodologies proposed by Elnemma et al.^82^ and Maharajan et al.^83^, respectively.

### Determination of the protein level

All results of the biochemical analyzes were expressed by “g of proteins” of the samples. In this case, the protein level was determined with a commercial kit (Bioténica Ind. Com. LTD, Varginha, MG, Brazil. CAS number: 10.009.00) based in the biuret reaction^84,85^. In general, Cu^2+^ ions, in an alkaline medium, react with the peptide bonds of proteins forming a blue complex specifically with protein, and the intensity of color, measured by ELISA plate reader at wavelength of 492 nm, is proportional to the protein concentration.

### Bioinformatics in silico analysis

The peptide structures PSPD2002 and PSPD2003 were modeled using the web server PEP-FOLD3 (https://bioserv.rpbs.univ-paris-diderot.fr/services/PEP-FOLD3/). Protein structures (targets) of Zebrafish organism (*Danio rerio*) catalase and superoxide dismutase was obtained by the homology construction technique by the SWISS-MODEL server (https://swissmodel.expasy.org/) with structural similarity values between 87.14% and 99.8%. The validation of the structures was verified with the SAVES v.6.0 server (https://saves.mbi.ucla.edu/). For simulations of docking molecular AutoDock tools (ADT) v4.2 were used to prepare binders and targets and AutoDock Vina 1.1.2 to perform calculations^86^. The binding affinity and interactions between residues were used to determine better molecular interactions. The results were visualized using ADT and UCSF Chimera X^87^.

### Assessment of Neutrophil activation (CD62L shedding)

We analyzed neutrophil activation by evaluating the expression and shedding of membrane-bound L-selectin (CD62L) by flow cytometry as previously described with adaptations^88^. Briefly, 100 μL of peripheral blood from healthy subjects was incubated with PSPD2002 or PSPD2003 (10 µg/ml or 100 µg/ml) for 30 min at 37 °C and followed by incubation with LPS (10 µg/ml, when indicated) for 30 min. After incubations, the erythrocytes were lysed by adding 2 ml of RBC lysis solution (Qiagen), and the leucocytes were stained with anti-human CD62L, CD66b, and Viability stain 510 (BD Biosciences). The CD62L expression was evaluated in CD66b + cells (neutrophils) by flow cytometry.

### Human Cytotoxicity assay and Nitric oxide production

The Spike protein fractions cytotoxicity was performed in murine alveolar macrophage AMJ2-C11 (ATCC CRL-2456) by the Alamar Blue™ assay. Cells were cultured in DMEM medium (Dulbecco's Modified Eagle medium) supplemented with 10% fetal bovine serum, 2 mM glutamine, 100 μg/mL streptomycin, and 100 U/mL penicillin, and incubated at 37 °C, in an atmosphere of 5% CO2. To evaluate of cytotoxicity, cells were seeded in 96 well plates (5 × 104 cells/well) and incubated with the Spike protein fractions at concentrations of 1; 10; 30; and 100 μg/ml in final volume of 200 μL for 24 h in a 5% CO2 incubator at 37 °C. After 24 h, 20 μL of Alamar Blue solution was added and incubated for 4 h. The fluorescence signal was monitored through a multi-plate reader using excitation wavelength 530–560 nm and 590 nm emission wavelength. The fluorescent signal generated from the assay was proportional to the number of viable cells in the sample. The assay was performed in quadruplicate. For Nitric oxide production AMJ2-C11 cell lineage was seeded in a 96 well plate (2.5 × 105 cells/well) and incubated with the SCIE at concentrations of 1; 10; 30 and 100 μg/ml for 1 h. After 1 h of treatment, the cells were stimulated with LPS (1 μg/ml) and maintained in culture for 24 h in a 5% CO2 at 37 °C. The production of nitric oxide was determined in the supernatants by the Griess method. The reading was performed in a spectrophotometer with absorbance of 570 nm.

### Statistical analysis

The data is reported as the mean standard error of the mean (SEM) and was statistically analyzed by one-way ANOVA test and Tukey’s post hoc test was used for comparisons. The survival analysis was performed by the Kaplan–Meier curve and the log-Rank test. *p* values < 0.05 were considered significant. The data were determined using Graph Pad Prism 5.0 software (Graph Pad Prism Software Inc.).

## Supplementary Information

Supplementary Information.

## Data Availability

All data generated or analyzed during this study are included in this published article and its supplementary information files.
