# Author Correction: A novel insight on SARS-CoV-2 S-derived fragments in the control of the host immunity

**DOI:** 10.1038/s41598-023-39134-1

**Published:** 2023-07-25

**Authors:** Thais Sibioni Berti Bastos, André Guilherme Portela de Paula, Rebeca Bosso dos Santos Luz, Anali M. B. Garnique, Marco A. A. Belo, Silas Fernandes Eto, Dayanne Carla Fernandes, Fausto Klabund Ferraris, Leticia Gomes de Pontes, Tábata Takahashi França, Leonardo José Gil Barcellos, Flavio P. Veras, Pamela Bermejo, Giovanna Guidelli, Carla Maneira, Fellipe da Silveira Bezerra de Mello, Gleidson Teixeira, Gonçalo Amarante Guimarães Pereira, Bianca H. Ventura Fernandes, Paulo R. S. Sanches, Helyson Lucas Bezerra Braz, Roberta Jeane Bezerra Jorge, Guilherme Malafaia, Eduardo M. Cilli, Danilo da Silva Olivier, Marcos Serrou do Amaral, Renata J. Medeiros, Antonio Condino-Neto, Luciani R. Carvalho, Glaucia M. Machado-Santelli, Ives Charlie-Silva, Jorge Galindo-Villegas, Tárcio Teodoro Braga

**Affiliations:** 1 Department of Pathology, Federal University of Parana, Curitiba, Brazil; 2 Graduate Program in Biosciences and Biotechnology, Instituto Carlos Chagas, Fiocruz-Parana, Brazil; 3 Department of Cell Biology, Institute of Biomedical Sciences, University of São Paulo, São Paulo, Brazil; 4 Brasil University, Descalvado, São Paulo, Brazil; 5 Center of Excellence in New Target Discovery (CENTD) Special Laboratory, Butantan Institute, São Paulo, Brazil; 6 Center of Innovation and Development, Laboratory of Development and Innovation, Butantan Institute, São Paulo, Brazil; 7 Veterinarian, São Paulo, Brazil; 8 Department of Pharmacology and Toxicology, Oswaldo Cruz Foundation, FIOCRUZ, Rio de Janeiro, Brazil; 9 Laboratory of Human Immunology, Department Immunology, Institute Biomedical Sciences, University São Paulo, São Paulo, Brazil; 10 Laboratory of Fish Physiology, Graduate Program of Bioexperimentation, University of Passo Fundo, Santa Maria, Brazil; 11 Graduate Program of Pharmacology, Federal University of Santa Maria, Santa Maria, Brazil; 12 Center of Research in Inflammatory Diseases, Ribeirão Preto Medical School, University of Sao Paulo, Ribeirão Preto, São Paulo, Brazil; 13 Department of Pharmacology, Ribeirao Preto Medical School, University of São Paulo, Ribeirao Preto, São Paulo, Brazil; 14 Laboratório de Genômica e bioEnergia (LGE), Institute of Biology - Unicamp, Campinas, Brazil; 15 Department of Genomics, Faculty of Biosciences and Aquaculture, Nord University, Bodø, Norway; 16 Laboratório de Controle Genético e Sanitário, Diretoria Técnica de Apoio ao Ensino e Pesquisa, Faculdade de Medicina da Universidade de São Paulo, São Paulo, Brazil; 17 Instituto de Química, Universidade Estadual Paulista, Araraquara, SP Brazil; 18 Department of Physiology and Pharmacology, School of Medicine, Federal University of Ceará, Fortaleza, CE Brazil; 19 Biological Research Laboratory, Goiano Federal Institute, Urutai Campus, Urutaí, GO Brazil; 20 Integrated Sciences Center, Federal University of Tocantins, Araguaína, TO Brazil; 21 Institute of Physics, Federal University of Mato Grosso do Sul, Campo Grande, MS 79070‐900 Brazil; 22 Laboratory of Physiology, INCQS/Fiocruz Zebrafish Facility, Department of Pharmacology and Toxicology, National Institute for Quality Control in Health, Rio de Janeiro, Brazil; 23 Laboratory of Cellular and Molecular Biology, Department of Cell and Developmental Biology, Institute of Biomedical Science, University of Sao Paulo, University of São Paulo, São Paulo, Brazil; 24 Department of Pharmacology, University of São Paulo-ICB/USP, São Paulo, Brazil

Correction to: *Scientific Reports* 10.1038/s41598-023-29588-8, published online 17 May 2023

The original version of this Article contained an error in the Introduction, where a reference has been included incorrectly as a hyperlink.

The correct reference is listed below:

Galindo-Villegas J. The Zebrafish Disease and Drug Screening Model: A Strong Ally Against Covid-19. *Front. Pharmacol.* **11** (680). 10.3389/fphar.2020.00680 (2020).

In the Introduction,

“In addition to traditional host models, zebrafish (*Danio rerio*), an extremely versatile and attractive organism, have gained prominence in research 10.3389/fphar.2020.00680.”

now reads:

“In addition to traditional host models, zebrafish (*Danio rerio*), an extremely versatile and attractive organism, have gained prominence in research^20^.”

The reference has now been added to the Reference list as Reference 20. As a result, the subsequent references have been renumbered.

The original Article has been corrected.

