# Supplementary material for: A novel insight on SARS-CoV-2 S-derived fragments in the control of the host immunity
Source: Sci Rep. 2023 May 17;13:8060. doi: 10.1038/s41598-023-29588-8 (PMC10191404; doi:10.1038/s41598-023-29588-8)
Supplement: Supplementary file 1 — Supplementary Information. [file 41598_2023_29588_MOESM1_ESM.pdf]

ACE2 – peptides

Analysis

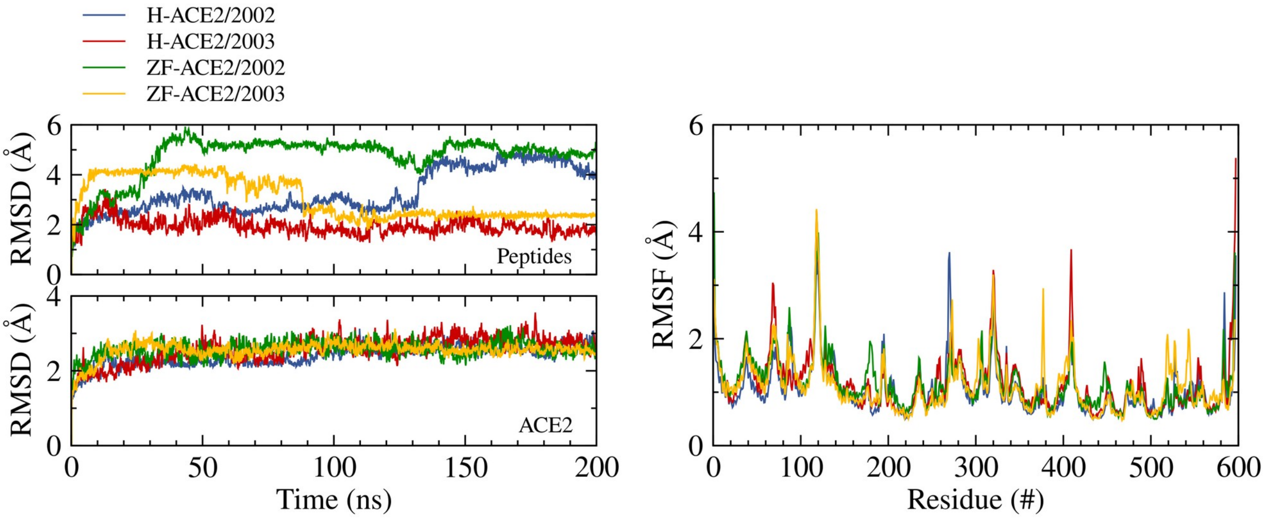

Fig 1 – Analysis of the stability of the simulated systems. Left – Root mean square deviation for the 2002 and 2003 peptides (top) and the human and zebrafish ACE2 proteases (bottom). Right – Root mean square fluctuation analysis for the ACE2 proteases

To access the convergence and stability of the simulations the RMSD and RMSF plots were done for all the systems (Fig 1). The RMSD plots for the peptides indicates a fluctuation over time depending on simulated system. 2002 peptide is bigger that 2003, and presented more fluctuations over time when compared to the shorter peptide, 2003. The same plot for the ACE2 of human and zebrafish presented the convergence of the proteases, indicating high stability over time. The RMSF plot shows the displacement of the residues of ACE2 proteases. Results for all the systems overlaps with minor intensity differences among them. The low values indicates the residues are stable and did not significantly change during the MD simulations, while the peaks indicates regions with poor secondary structure stability, like loops and turns.

The radius of gyration (RoG) and surface area completes the stability analysis showing that the proteases were stable over time. RoG shows if the proteases increased or decreased its size over time. For all the simulations the RoG were around 25.3 *Å*, indicating stability (Fig S1 - left). The surface area of the proteases were also stable over time, with minor fluctuations for the zebrafish/2003 complex (Fig S1 – right).

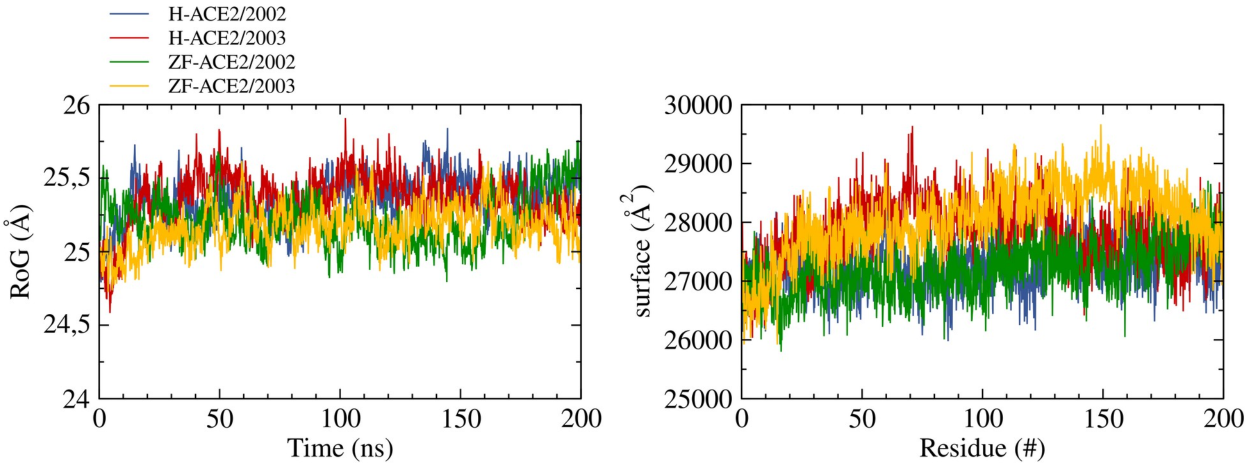

Fig S1 – Radius of gyration and Surface area for the ACE2 proteases. Left – Radius of gyration for the human and zebrafish in the presence of peptides 2002 and 2003. Right – Solvent-accessible surface area for the simulated complexes of human and zebrafish ACE2 proteases in the presence of peptides 2002 and 2003.

MM/GBSA energy calculation were done over the last 100 ns of simulation. The average binding value were -48.3 kcal/mol, -44.9 kcal/mol and -45.0 kcal/mol for the H-ACE2/2002, H-ACE/2003 and ZF-ACE2/2002 respectively (Fig 2). For the zebrafish ACE2 in the presence of the peptide 2003, the energy value was around -17.3 kcal/mol.

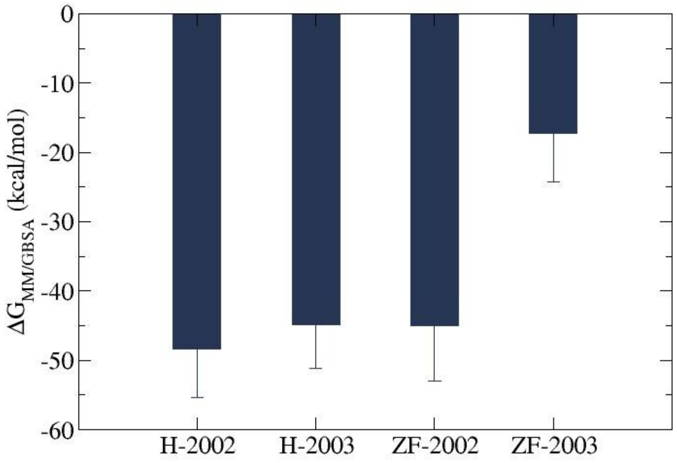

Fig 2 – Binding energy for the peptides 2002 and 2003 in the presence of the human and zebrfish ACE2 proteases.

The energy decomposition indicated that the peptides were in the same region for the systems H-2002, H-2003 and ZF-2002, while it was slightly different for the ZF-2003. (Fig 3 or S2). The protease residues with negative values cooperates to bind the peptide, while the positive values indicate the protease residue does not cooperate to the binding. For the first three complexes the peptide bound in similar regions: between residues 60-100, 160-200 and 360-400. In these cases, the contribution of the protease residues changed in intensity and also the residues that were contributing to the binding. On the other hand, the ZF-2003 complex only shares the region of the residues between 160-200, with another contribution around the residues 480-500. This could be the reason for the large total binding difference between this system compared to the others. Representative structures for each simulated system shows the peptides interacting with the human and zebrafish ACE2 protease (Fig S3).

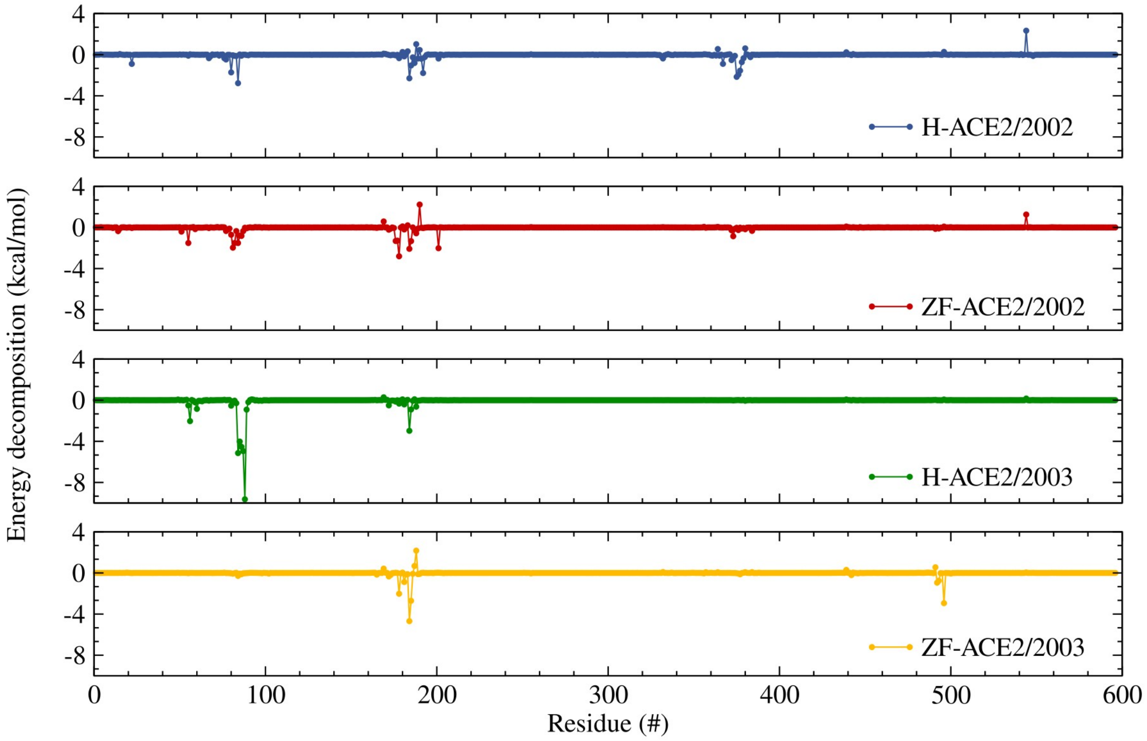

Fig 3 or S2 – Binding energy decomposition per residue obtained with MM/GBSA for the last 100 ns and all the four complexes.

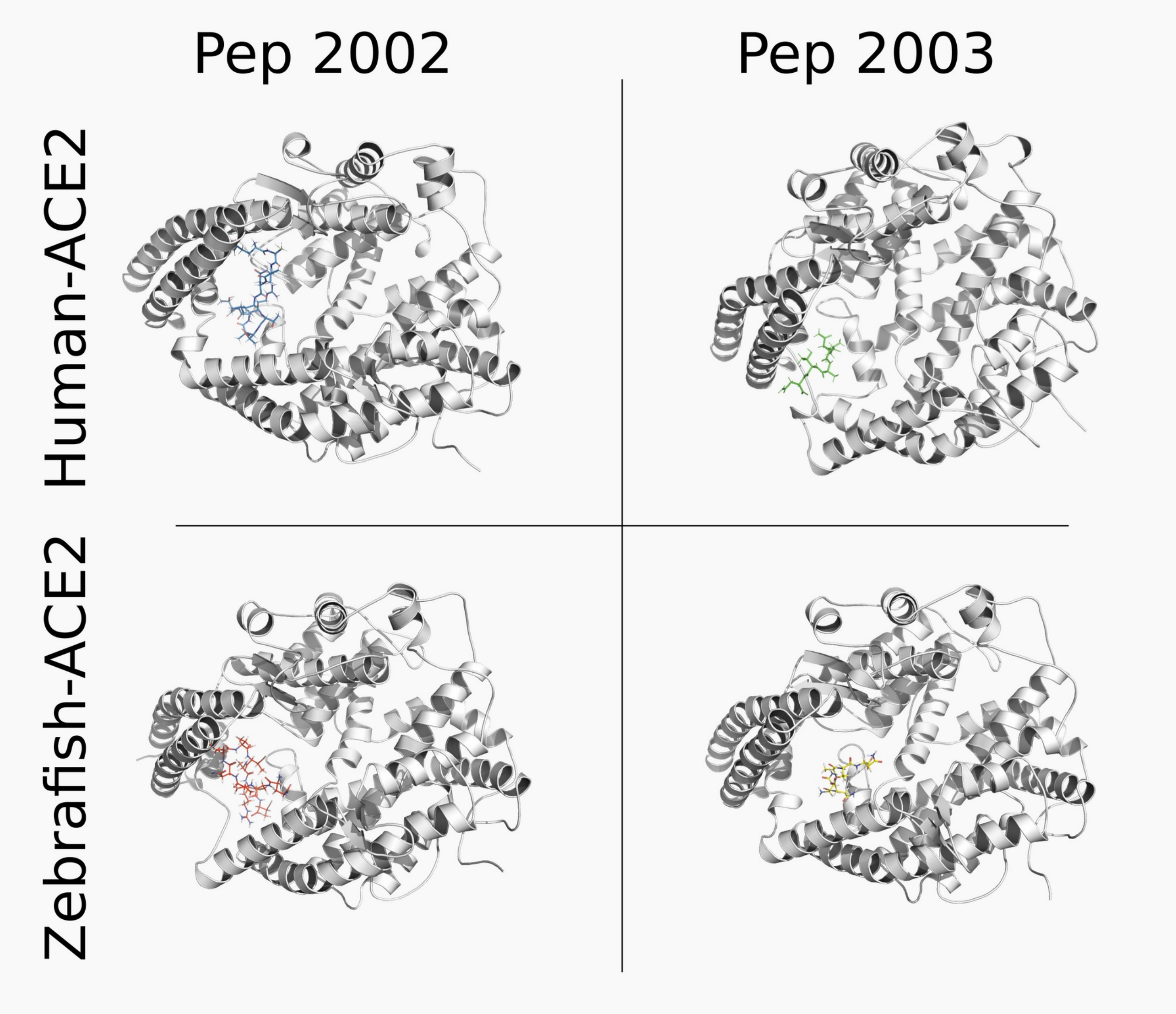

Fig S3 – Representative structure for the peptides 2002 and 2003 in the presence of the human and zebrafish ACE2 proteases.
